# Impact of hypoxic versus oxic conditions on local tumor control after proton irradiation in a rat prostate carcinoma

**DOI:** 10.1016/j.ctro.2025.100957

**Published:** 2025-04-12

**Authors:** Michaela Schmitt, Christin Glowa, Ina Kurth, Peter Peschke, Stephan Brons, Christian P. Karger

**Affiliations:** a German Cancer Research Center (DKFZ) Heidelberg, Division of Medical Physics in Radiation Oncology, Germany; b Faculty of Biosciences, Heidelberg University, Germany; c Heidelberg Institute for Radiation Oncology (HIRO) and National Center for Radiation Research in Oncology (NCRO), Heidelberg, Germany; d University Hospital Heidelberg, Department of Radiation Oncology and Radiotherapy, Germany; e German Cancer Research Center (DKFZ) Heidelberg, Division of Radiooncology Radiobiology, Germany; f Heidelberg Ion Beam Therapy Center (HIT), Heidelberg, Germany

**Keywords:** Proton radiotherapy, Dose-response curves, Hypoxia, Oxygen enhancement ratio, Relative biological effectiveness

## Abstract

**Background and purpose:**

Hypoxia in tumors significantly contributes to radiation resistance, often leading to poor treatment outcomes. *In vitro* studies demonstrated that proton irradiation not only exhibits an increased relative biological effectiveness (RBE), but also a lower oxygen enhancement ratio (OER). This study explored the impact of hypoxic and oxic conditions on local tumor control after proton irradiation in a rat prostate carcinoma.

**Material and methods:**

Subcutaneously transplanted Dunning R3327-HI rat prostate carcinomas were irradiated with varying single doses of protons under oxic or hypoxic conditions. Hypoxia was induced by clamping the tumor-supplying vessels. The biological endpoint was local tumor control assessed 300 days after irradiation. Dose-response curves were determined and based on the doses required for 50 % tumor control probability (TCD_50_), the RBE and OER were calculated.

**Results:**

The TCD_50_ value was significantly higher under hypoxic than under oxic conditions (73.4 ± 1.9 Gy vs. 50.5 ± 1.6 Gy), resulting in an OER of 1.45 ± 0.06 for proton irradiation. Compared to photon irradiation, the RBE for protons was of 1.23 ± 0.07 under oxic and 1.30 ± 0.04 under hypoxic conditions.

**Conclusion:**

Proton irradiations showed a 5 % reduction in OER compared to the previously measured photon value of 1.53 ± 0.08, suggesting a slightly higher effectiveness of protons in hypoxic tumors as compared to photons.

## Highlights

• Clamping reversibly inhibits tumor perfusion in rat prostate tumors.
• Under hypoxic conditions, protons were slightly more effective than photons.
• The oxygen enhancement ratio of protons was 5 % lower than for photons.
• The relative biological effectiveness of protons was 6 % higher under hypoxia.

## Introduction

Most solid tumors exhibit spatial and temporal variations in oxygen levels, leading to areas with oxygen deficiency (hypoxia). This altered microenvironment can trigger various cancer hallmarks and is associated with increased tumor aggressiveness, resulting in poor treatment prognosis [1]. Tumor hypoxia can arise from limited oxygen diffusion due to abnormal spatial distribution of the vascular structure, known as chronic hypoxia. While chronic hypoxia lasts longer than 24 h, acute hypoxia, also termed as perfusion-limited hypoxia arises due to fluctuating vasoconstrictions and is only present for a few minutes to hours [1,2]. Hypoxic tumor cells show enhanced resistance to radiation, posing a significant challenge in radiation therapy. This may be explained by the oxygen fixation hypothesis, which proposes that radicals produced by ionizing radiation are oxidized and thereby fix the DNA damage [3]. The impact of the oxygen level on the radiation effectiveness is quantified by the oxygen enhancement ratio (OER), being the ratio of doses under hypoxic (D_hypox_) and oxic (D_ox_) conditions leading to the same biological effect. In clinical practice, the complex impact of hypoxia on radiobiological response presents a major treatment challenge [4].

Particle irradiation is a promising strategy to treat tumors that are radioresistant to conventional photon irradiation. The inverted depth dose profile with low doses in the entrance region and a maximum dose at the end of range (Bragg peak) allows for highly conformal targeting of the tumor [5]. Superimposing multiple Bragg peaks results in a spread-out-Bragg peak (SOBP), which is used to deliver a therapeutic dose throughout the entire tumor while sparing the surrounding healthy tissue. In addition, particles exhibit an increased linear energy transfer (LET) leading to an enhanced relative biological effectiveness (RBE), which is, among other factors, clearly correlated with an increased proportion of clustered DNA damage [[6], [7], [8]]. It has been shown in three different sublines of the Dunning R3327 rat prostate carcinoma, that the doses required for local tumor control after carbon ion irradiation are much less dependent on the individual biological characteristics of the tumor sublines compared to photons [9]. Given that the investigated tumor sublines also exhibited distinct variations in their hypoxic fraction, it was hypothesized that the impact of hypoxia on the radiation response would decrease significantly for high-LET radiation, e.g carbon ions. In this context, a previous study demonstrated that clamping-induced hypoxia results in a smaller increase in tumor control doses for carbon ions than for photons [10]. This finding indicated a lower OER for carbon ions relative to photons, and in turn, a higher RBE under hypoxic than under oxic conditions.

While these findings clearly showed that carbon ions can counteract the impact of hypoxia on radiation resistance compared to photon irradiation, it remained unclear if there is a similar, but potentially less pronounced effect for protons. While this might be questioned due to the considerably lower proton LET, it is important to note that the LET is still enhanced relative to the LET of secondary electrons released by photon beams [5,11]. This suggests that protons may still exhibit differential biological effects relative to photons, particularly expressed by the OER. To the authors’ knowledge, only a limited number of *in vitro* studies [[12], [13], [14]] and even fewer *in vivo* studies have compared the effectiveness of protons in tumors under oxic and hypoxic conditions systematically in terms of OER, while employing independent treatment arms for photon and proton irradiation [15].

Based on 10 % survival data in Chinese hamster cells (V79), Prise et al. found an OER range for protons of 1.89 to 2.77, depending on the mean energy, which was significantly lower than the X-ray OER of 3.14, and hence hypoxia-induced radiation resistance was less for protons than for photons [14]. In contrast, Raju et al. reported no significant difference in OER between photons and protons for the same biological endpoint and cell line [12]. In an *in vivo* study, Urano et al. assessed the colony formation of irradiated C3Hf/Sed mouse fibrosarcoma (FSa-II) cells in the lung as the biological endpoint, and found an average RBE of 1.16 and no significant differences in OER between photons and protons [15]. Consequently, the existing data did not show a clear trend regarding the effectiveness of proton irradiation under hypoxic conditions relative to photons and none of these studies accounted for the long-term interaction with the tumor microenvironment, the host immune system, or the therapeutic outcome. Hence, there is a need to further investigate the potential radiobiological advantages of proton therapy under hypoxia.

To address these gaps, we conducted proton dose–response experiments using the rat prostate carcinoma Dunning R3327-HI in male Copenhagen rats under both oxic and hypoxic conditions using local tumor control at 300 days after irradiation as biological endpoint, with the aim to determine OER and RBE values. To achieve loco-regional and temporary hypoxia, blood flow was suppressed by clamping the tumor-supplying vessels shortly before and during irradiation. This technique allows for the investigation of tumor radiation response without the sensitizing effect of oxygen caused by radiation-induced reactive oxygen species (ROS) and subsequent DNA damage fixation, while keeping the animal alive [3]. In addition, the short-term effects following clamping release and its reversibility with respect to the tumor microenvironment were investigated.

## Material and methods

### Animals and tumor model

Male Copenhagen rats (weight: 283 ± 40 g, age: 16 ± 5 weeks; bred in-house) were subcutaneously implanted with the syngeneic Dunning prostate carcinoma R3327-HI. Under inhalation anesthesia with a mixture of 2.5 % isoflurane (CP-Pharma Handelsgesellschaft mbH, Germany) and oxygen at 2 l/min, a small skin incision of approximately 4 mm was made laterally to the third distal part of the right hind leg to create a small subcutaneous pocket. The fresh tumor fragment from a donor tumor was inserted and the pocket was closed with a 9 mm metal clip (BD AutoclipTM, Becton Dickson GmbH, Germany). Donor tumors were grown from a cryopreserved stock [16,17]. The animal facility and experiments were approved by the German animal welfare authority and the local ethics committee in accordance with Directive 2010/63/EU of the European Parliament and Council on the protection of animals used for scientific purposes (reference number: 35–9185.81/G-197/17 and 35–9185.81/G-151/22, Karlsruhe, Germany). Animals were housed under standard laboratory conditions.

### Irradiation and follow-up

This study used the same experimental setup as in previous studies with photon and carbon ion irradiation [10,16]. A total of 111 tumors were irradiated with single fractions of protons at increasing dose levels under either oxic or hypoxic conditions (Table 1). Animals were randomly assigned to the different dose groups once their tumor reached a mean diameter of 10.5 mm (range: 7.0 – 12.5 mm).

**Table 1:** Proton dose levels and number of irradiated animals.

Experiment	Dose levels [Gy] (animals per dose level)	Total number of animals
Protons (oxic)	40 (6), 44 (6), 48 (6), 52 (6*), 56 (6), 60 (6), 64 (6), 68 (6), 72 (6)	54
Protons (hypoxic)	50 (6), 55 (6), 60 (6#), 65 (6), 70 (6), 75 (6), 80 (6$), 85 (5), 90 (5), 95 (5#)	57
**Total**		**111**

* One animal was euthanized due to overall bad condition.

# One animal was euthanized due to weight loss.

$ One animal died for unknown reason.

**Fig. 1:**
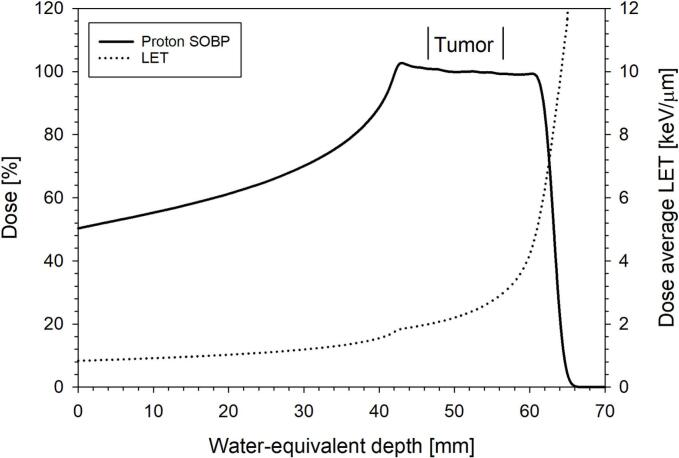
Tumor position in the proton depth dose (solid line) and LET (dashed line) distribution.

Proton irradiation was performed at the Heidelberg Ion Beam Therapy Center (HIT) using the active raster scanning technique [18]. The anesthetized rats (mixture of 2.5 % sevoflurane (SevoFlo®, Zoetis, Belgium) and oxygen at 2 l/min) were fixed and the tumors were positioned at the center of a 20 mm SOBP having a lateral field size of 18 x 18 mm^2^ (Fig. 1). To induce hypoxia in the tumor, the tumor-supplying vessels were clamped 10 min before and 5  min during irradiation (see details in [10]). The mean dose-average LET in the tumor was 2.4 keV/µm (range: 2.0 – 3.0 keV/µm) (Fig. 1).

During follow-up, tumor volumes were measured once per week with a caliper. The biological endpoint of this study was local tumor control after 300 days, defined as the absence of detectable tumor growth.

### Quantitative analysis

For the selected biological endpoint, dose–response curves were fitted to the actuarial tumor control rates using the logistic dose–response model along with the maximum likelihood fitting procedure of the software STATISTICA (version 10.0, Statsoft Inc., https://www.statsoft.com). The doses at 50 % local tumor control (TCD_50_) probability were determined from the fit parameters and the OER was calculated as the ratio of TCD_50_ under oxic and hypoxic conditions. Adding the photon data from the previous study [10], the RBE under oxic and hypoxic conditions was determined as the ratio of the respective TCD_50_ values for photons and protons.

### Statistics

The effective sample size method was used to account for animals with incomplete follow-up in the fitting procedure by correcting the number of treated and responding tumors to match actuarial response rates and their variances [19]. The standard errors (SE) of TCD_50_, OER, and RBE were calculated by error propagation and for TCD_50_, the correlation of the fit parameters was considered by their covariance. The 90 % confidence intervals (CIs) were calculated using Fieller’s Theorem [20]. Statistical differences of TCD_50_, OER and RBE values were assessed using a one-sided Z-Test at a significance level of p ≤ 0.05.

### Immunohistochemistry

Immunohistochemical staining was performed to investigate potential short-term effects of the clamping procedure on the tumor microenvironment. For this purpose, 5 unirradiated animals each served as controls without and with clamping of the tumor supplying vessel (without release). Additionally, 4 animals each were clamped and sacrificed either 5 or 60 min after clamping was released. The clamping procedure was timed to 15 min.

The perfusion marker Hoechst 33342 (15 mg/kg, Merck KGaA, Germany) was injected into the right ventricle of the rats’ heart 30 s before tumor dissection. Immediately afterwards the tumors were cleaned with PBS and embedded in Tissue-Tek® O.C.T. Compound (Sakura Finetek Germany GmbH, Germany) on dry ice and stored at −80 °C. Afterwards, three cryosections of 7 µm of different tumor depth were cut and fixed with methanol and acetone at 4 °C. Immunofluorescence staining was performed to detect CD31 as marker for the endothelial vascular structure, Ki-67 as proliferation marker and carbonic anhydrase 9 (CA9) as endogenous hypoxia marker. Thawed slides were blocked against non-specific bindings with Protein block (Agilent Technologies, Germany) for 60 min at room temperature (RT). The primary antibodies against HIF-1α (1:200, NB100-449, Novus Biolgicals), CD31 (1:1000, #AF3628, R&D Systems) and Ki-67 (1:1000, ab16667, Abcam) were each diluted in 5 % BSA in 1x PBS and incubated overnight at 4 °C in a humidified chamber. The primary antibody CA9 (1:1000, NB100-417, Novus Biologicals) was diluted similarly and incubated for 2  h at RT in a humidified chamber. After rinsing the slides with PBS, they were incubated with the corresponding secondary antibody conjugated to Alexa555 or Alexa647 (#A21432, #A21429, #A32795 ThermoFisher Scientific) for 1 h at RT in the dark. After washing with PBS, the Ki-67 and CA9 stained slices were counterstained with DAPI (100  ng/ml, Carl Roth GmbH + Co. KG) for 3 min. All sections were mounted using Fluoromount- G^TM^ mounting medium (Invitrogen). In addition, Hematoxylin & Eosin (HE) staining (Carl Roth, Karlsruhe, Germany) was performed on cryosections [9]. Images of the whole tumor section were acquired with a 20x magnification using the Axio Scan.Z1 microscope (Carl Zeiss Microscopy GmbH, Germany). QuPath Version 0.5.0 was used for bioimage analysis [21]. Based on Hoechst or DAPI staining, a pixel classifier was used for tissue detection after manually removing artefacts. Cell detection was then performed based on DAPI or Hoechst staining. In five annotations of 1000 × 1000 µm^2^ representing the image set at least 100 cells were classified to train an object classifier for each channel and to quantify the amount of positively stained cells. The diffusion distance was determined as the mean of the 95 % smallest distances of Hoechst^+^ cells from their nearest Hoechst^+^ CD31-labeled vessel. Statistical differences were tested using the SigmaPlot Version 14.0. A one-way analysis of variance (ANOVA) was conducted, followed by post-hoc comparisons using the Holm-Sidak test. P ≤ 0.05 was considered as statistically significant.

## Results

**Fig. 2:**
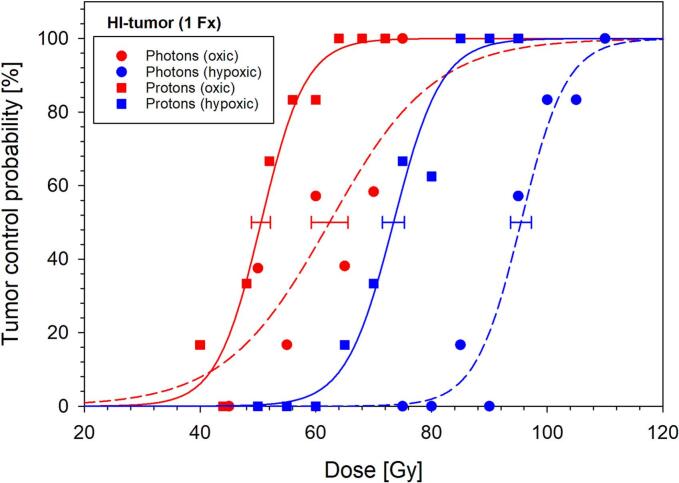
Dose-response curves for the biological endpoint local tumor control 300 days after proton irradiation under oxic (red) and hypoxic (blue) conditions. The proton data (rectangles) is compared with photon data (circles) acquired previously under identical conditions [10]. The increase in TCD_50_ values of protons relative to photons as well as hypoxic relative to oxic conditions was significant (p ≤ 0.05). Error bars represent the single standard error of TCD_50_ [10]. (For interpretation of the references to colour in this figure legend, the reader is referred to the web version of this article.)

**Fig. 3:**
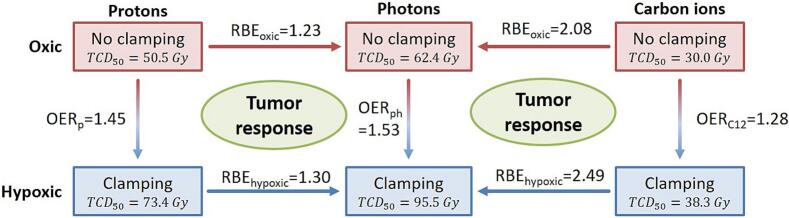
Relation of the TCD_50_, OER and RBE values for protons (p), photons (ph) and carbon ions (C12) under oxic (red) and hypoxic (blue) conditions, measured in this and in a previous study [10]. OER decreases from photons over protons to carbon ions, leading to RBE values that are higher under hypoxic than under oxic conditions. (For interpretation of the references to colour in this figure legend, the reader is referred to the web version of this article.)

To determine the effects of protons, tumors were irradiated with protons either under oxic (54 animals) or hypoxic conditions (57 animals) (Table 1). Under both conditions, 100 % local tumor control could be achieved. Under hypoxia, the dose–response curve was shifted to significantly higher doses (Fig. 2) compared to oxic conditions. The resulting TCD_50_ values were 73.4 ± 1.9 Gy under hypoxic and 50.5 ± 1.6  Gy under oxic conditions, resulting in an OER of 1.45 ± 0.06 for protons (Table 2). Including previously published photon data, the RBE was 1.23 ± 0.07 under oxic and 1.30 ± 0.04 under hypoxic conditions. While the differences of the TCD_50_ values differed significantly between photons and protons as well as between oxic and hypoxic conditions (p ≤ 0.05), this was not the case for the OER and RBE values. In contrast to photons, the slopes of the proton curves were similarly steep under oxic and hypoxic conditions and comparable to the slope of the hypoxic photon curve (Fig. 2). Fig. 3 illustrates the relation between the measured TCD_50_, RBE and OER values of both oxygen conditions in comparison to the photon and carbon ion irradiation from the previous study.

**Table 2:** TCD_50_ values for protons and photons under oxic and hypoxic conditions together with the resulting OER and RBE values. Uncertainty is provided as single standard error (SE) and 90 % confidence intervals (CI). The differences of TCD_50_ between protons and photons and between hypoxic and oxic conditions were signigicant (p ≤ 0.05), while this was not the case for OER and RBE values. The photon data were acquired under identical conditions in a previous study [10].

	TCD_50_ ± SE (90 % CI) [Gy]		RBE ± SE (90 % CI)
	Photons [10]	Protons	
Oxic	62.4 ± 3.2 (56.6–69.8)	50.5 ± 1.6 (47.4–53.4)	1.23 ± 0.07 (1.12–1.36)
Hypoxic	95.5 ± 1.8 (92.2–98.9)	73.4 ± 1.9 (70.0–77.0)	1.30 ± 0.04 (1.23–1.37)
OER ± SE (90 % CI)	1.53 ± 0.08 (1.40–1.68)	1.45 ± 0.06 (1.36–1.56)

**Fig. 4:**
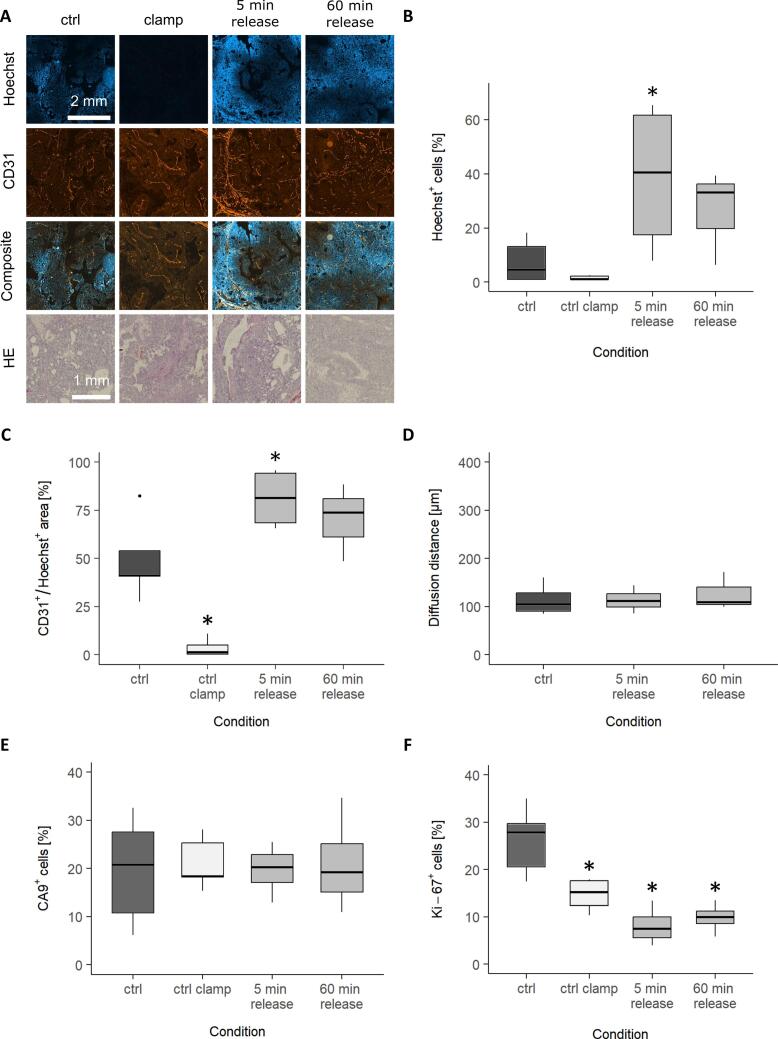
Histological evaluation of the effect of the clamping procedure in the Dunning R3327-HI prostate carcinoma comparing unclamped tumors (dark grey), clamped tumors (white) and tumors after 5/60  min clamping release (light grey). **(A)** Representative immunofluorescence stainings for perfusion (Hoechst in blue) and vessels (CD31 in red) and HE staining for non-clamping, clamping, 5 and 60 min after clamping release. **(B)** Perfusion (Hoechst^+^ cells) and **(C)** amount of perfused vessels (CD31^+^/Hoechst^+^ vessels) were strongly decreased during the clamping process, while it was increased 5 and 60 min after clamping release with a trend back to the pre-clamping state. **(D)** The diffusion distance, determined as the mean of the 95 % smallest distances of Hoechst^+^ cells from their nearest Hoechst^+^ CD31-labeled vessel, was not affected by the clamping procedure. **(E)** The amount of CA9^+^ cells was not influenced by clamping the tumor-supplying vessels. **(F)** Ki-67 was significantly reduced during clamping and after clamping release. For E & F, representative images can be found in the supplementary Figs. 1 and 2. Data is expressed as median values with interquartile ranges (IQR). Whiskers represent the range within 1.5 times the IQR, and outliers (beyond 1.5 times the IQR) are presented as points. *p ≤ 0.05 vs. ctrl. (For interpretation of the references to colour in this figure legend, the reader is referred to the web version of this article.)

To confirm the perfusion reduction of the clamping procedure and the reversibility of the tumor micromilieu changes, immunohistochemical analyses of the tumor samples were performed before, during clamping and shortly after the clamping was released. During clamping the amount of Hoechst^+^ cells and Hoechst^+^ CD31-labeled vessels (Fig. 4A-C) decreased to nearly zero. After clamping release, a significant increase of Hoechst^+^ cells relative to the unclamped control was observed with a trend back to the pre-clamped state over the observed period of 5 to 60 min. The oxygen diffusion distance did not change (Fig. 4D). The clamping procedure had no lasting effect on the tumor structure as confirmed by the HE staining (Fig. 4A). Nor did it affect the amount of CA9^+^ cells (Fig. 4E, Supplementary figure 1) or induce the translocation of HIF-1α to the cell nuclei (Supplementary figure 2). However, clamping induced a significant decrease in Ki-67^+^ cells (Fig. 4F, Supplementary figure 3).

## Discussion

The higher dose conformity of protons compared to photons resulted in a rapidly growing number of proton therapy centers worldwide [22]. To further improve clinical outcomes, ongoing research is focused on optimizing the physical aspects of treatment planning and delivery as well as on a better understanding of the biological effects of protons. As tumor hypoxia contributes significantly to radiation resistance, this study investigated the impact of varying oxygen supply of solid tumors on the proton radiation response.

It has recently been demonstrated for the Dunning R3327-HI rat prostate carcinoma that high-LET carbon ions are significantly more effective in hypoxic tumors than photons [10]. While protons are generally considered as low-LET radiation, their LET is still higher than that of secondary electrons released by photons and therefore, differences in the biological response may be expected. Using the previously established experimental setup [10], dose-response curves for the endpoint local tumor control after proton irradiation were acquired under oxic and hypoxic conditions. Hypoxic conditions were induced by clamping the tumor supplying vessels 10 min before and during irradiation, thereby blocking the vascular perfusion, nutrient supply and inducing acute ischemic hypoxia. As an alternative technique to induce hypoxic conditions, other studies have exposed the animals to 100 % nitrogen during irradiation. This, however, led to the animal’s death and tumor radiation response could therefore only be evaluated *in vitro* by clonogenic cell survival assays after removing the tumor [23,24]. To demonstrate that the only impact of the clamping procedure on the tumor microenvironment was the blockage of perfusion and the induction of acute hypoxia during clamping, an additional histological study was conducted investigating the effects following clamp release.

Double staining for the vascular marker CD31 and the perfusion marker Hoechst showed an almost complete absence of perfused vessels and Hoechst^+^ cells during the clamping procedure, demonstrating the complete blockage of the perfusion in the tumor. To ensure that the clamping-induced perfusion blockage did not permanently alter the vascular structure and function, tumors were extracted 5 and 60  min after clamping release. For the these time points, an increase in perfused vessels and Hoechst^+^ cells was observed as compared to the unclamped control indicating a rapid vascular reperfusion. Since the diffusion distance was not affected by the clamping procedure, damage to the vascular structure appears unlikely. Instead, the increased amount of perfused vessels and Hoechst^+^ cells may be explained by the high intravascular pressure induced by the clamping procedure, leading to perfusion of immature or previously occluded vessels.. These findings are also consistent with the results of a photoacoustic study in the same tumor model, which showed a strong decrease in oxygen saturation during clamping and a complete reperfusion after clamping release [25]. In contrast to the histological results, Denekamp et al. found a short-term reduction in blood flow using the ^86^Rb extraction technique 15 min after 30 min of clamping, however, the authors also observed a recovery to normal levels within 24 h [26]. Another study that used exactly the same technique as this study, did not detect any changes in Hoechst^+^ cells and the CD31-labeled vascular structures 24 h after clamping release [10]. It might therefore be hypothesized that the observed perfusion increase is only transient and does not affect the outcome of the dose-response studies, as no long-lasting changes in vascular structure or perfusion were observed in the tumor. This confirms that 15 min clamping is suited to reversibly induce acute hypoxia.

While the photoacoustic study by Bendinger et al. already demonstrated that clamping the tumor-supplying vessels significantly reduced oxygen saturation in the already moderately hypoxic HI-tumor [25], the present histological study aimed to assess the potential response of tumor cells to the oxygen deprivation and its reversibility. Exogenous markers (e.g. pimonidazole) were not used as they bind covalently and therefore changes in hypoxia could not be detected after clamping release [27], instead endogenous markers were used. The expression level of CA9 and HIF-1α were not affected during or after clamping. Nor was the translocation of HIF-1α to the cell nuclei affected, which is the initial cellular response to hypoxia [4]. This might be explained by the short duration of the additional hypoxic period of 15 min, which may not be sufficient to trigger the hypoxic cell response.

Only a few studies investigated clamping-induced changes in tumor growth and cell proliferation. Devi et al. reported no significant effect on tumor growth after clamping for 1 h, whereas Denekamp et al. demonstrated a regrowth delay for clamping durations of 2–8 h. Clamping for 18  h even resulted in no tumor regrowth [26,28]. Since the clamping time was only 15 min, which is much shorter, no growth delay would be expected. Nevertheless, a cell proliferation reduction shortly after clamping release was detected. This finding may be considered as a short-term effect as other studies have shown no change in proliferation 24 h after 15 min of clamping [10].

Regarding tumor control, this study demonstrated that protons have a higher biological effectiveness than photons. This is indicated by RBE values greater than one under both oxic and hypoxic conditions. However, the OER of protons was only slightly lower than for photons (1.45 ± 0.06 vs 1.53 ± 0.08), indicating a slightly higher or comparable efficacy in hypoxic tumors. In contrast, carbon ions exhibited an OER of 1.28 ± 0.08, making them significantly more effective under hypoxic conditions than photons and protons (Fig. 4) [10]. This finding aligns well with the *in vitro* data-based OER vs LET model of Wenzl and Wilkens et al., who reported a slow decrease in OER at low and a rapid decrease at high-LET [29]. The correlation between decreasing OER and increasing LET can be explained by the increasing ionization density that leads to clustered DNA damage for high-LET particles, which are more frequently produced by direct effects rather than indirect effects via free radicals. While free radicals such as ROS can fix DNA damage and thereby prevent DNA repair, complex clustered DNA lesions such as those produced by high-LET particles are difficult to repair, even without fixation by ROS [30]. The response of tumors to high-LET radiation is therefore considered less dependent on the oxygen level. In contrast, the response to low-LET irradiation relies predominantly on ROS-induced indirect effects [[31], [32], [33], [34]] and therefore, a higher oxygen dependence and thus higher OER-values are expected. In this regard, this study showed that the biological effects of protons are more similar to photons than to carbon ions.

*In vitro* OER values between 2.6 and 2.9 have been reported for proton irradiation [[12], [13], [14],^35^]. In contrast, *in vivo* OER values are generally lower due to the abnormal tumor microenvironment. To achieve best-possible tumor oxygenation, animals were breathing 100 % oxygen during irradiation. However, complete tumor oxygenation may not be achieved because of impaired vascular structure and its limited perfusion [36], partially leading to irreversible chronic hypoxia in unclamped tumors. This may explain the lower OER value of 1.53 compared to the published *in vitro* values greater than 2. To the authors’ knowledge, only Urano et al. performed a preclinical study comparing photon against proton treatments under oxic and hypoxic conditions and hence determining OER and RBE values for both modalities and oxygenation conditions, respectively. In this study, lung colony formation of FSa-II tumor cells were investigated 13 days after irradiation under oxic (whole thorax irradiation) and hypoxic (tumor irradiation in ectopic position) conditions. It should be noted, however, that ectopic tumor positions might not be sufficient to induce hypoxia. This might explain why no difference between oxic and hypoxic conditions were detected neither after photon nor proton irradiation [15], while we found an OER value of 1.45 for protons. Under hypoxic conditions, this indicates a slightly higher effectivness of protons relative to photons exhibiting an OER of 1.53 [10]. In line with this study’s results, Urano et al. measured a mean RBE value of 1.16 ± 0.12 showing the slightly higher effectiveness of protons compared to photons [15].

This study could additionally show that the response to proton irradiation is less dependent on intratumoral heterogeneity, as the slopes of the dose–response curves were steeper than for photons under oxic conditions [9]. Since the slope of the photon curve under hypoxic conditions was comparable to that of the proton curves, it is hypothesize that the heterogeneity underlying the oxic photon curve is a result of spatially varying oxygen levels. Similar findings were obtained in the previous carbon ion study [10]. It has to be noted that this study is the first full *in vivo* study considering the long-term effect of the tumor micro milieu as well as the clinically relevant endpoint local tumor control after a long-term follow-up of 300 days.

While the OER values between *in vitro* and *in vivo* experiments differ significantly, these differences are not as high for RBE values. Paganetti et al. reported a mean RBE of 1.22 ± 0.02 for 65–250 MeV proton irradiations for *in vitro* systems and 1.10 ± 0.01 for *in vivo* systems [37]. Based on early experiments, the International Commission on Radiation Units and Measurements (ICRU) recommends a generic proton RBE of 1.1 [38]. Although there is an ongoing discussion whether a variable RBE model is clinically required to account for the experimentally established RBE increase at the distal SOBP [39], this is not expected to play a role in this study as longitudinal safety margins of several millimeters were used.

Recent data even suggested a slightly higher average proton RBE of 1.15, nevertheless, the clinically adopted value of 1.1 seems reasonable to avoid underdosage in the tumor [40,41]. The measured RBE value of 1.23 ± 0.07 is slightly higher than the fixed proton RBE, however, the value of 1.15 is still included in the confidence interval. Additionally, the tumor subline Dunning R3327-HI already expresses a modest fraction of hypoxic cells leading to a slightly higher radiation resistance for photons than for protons and thus to an increased RBE. Besides this, also other factors such as biological endpoint, LET and fractional dose may play a role.

While the present proton as well as the previous carbon ion study [10] investigated the impact of hypoxia on the radiation response for a clinical relevant endpoint, they did not consider the influence of fractionation as it has been done for carbon ions under unclamped conditions [[42], [43], [44]]. If reoxygenation during a fractionated treatment course differs between photons, protons and carbon ion, the respective OER values and their proportions may differ as well.

In summary, this study found that the OER of protons is 5 % lower than for photons indicating a 6 % higher proton RBE under hypoxic as compared to oxic conditions. Considering that carbon ions counteract hypoxia even more effectively, this indicates a clear decrease in OER with increasing LET.

Funding statement

This work was funded by the 10.13039/501100001659Deutsche Forschungsgemeinschaft (DFG, German Research Foundation − KA 2679/3-2 and GL 893/1-2).

## Declaration of competing interest

The authors declare that they have no known competing financial interests or personal relationships that could have appeared to influence the work reported in this paper.
