# Supplementary material for: Impact of hypoxic versus oxic conditions on local tumor control after proton irradiation in a rat prostate carcinoma
Source: Clin Transl Radiat Oncol. 2025 Apr 12;53:100957. doi: 10.1016/j.ctro.2025.100957 (PMC12223568; doi:10.1016/j.ctro.2025.100957)
Supplement: Supplementary Data 1 [file mmc1.pdf]

Supplementary material

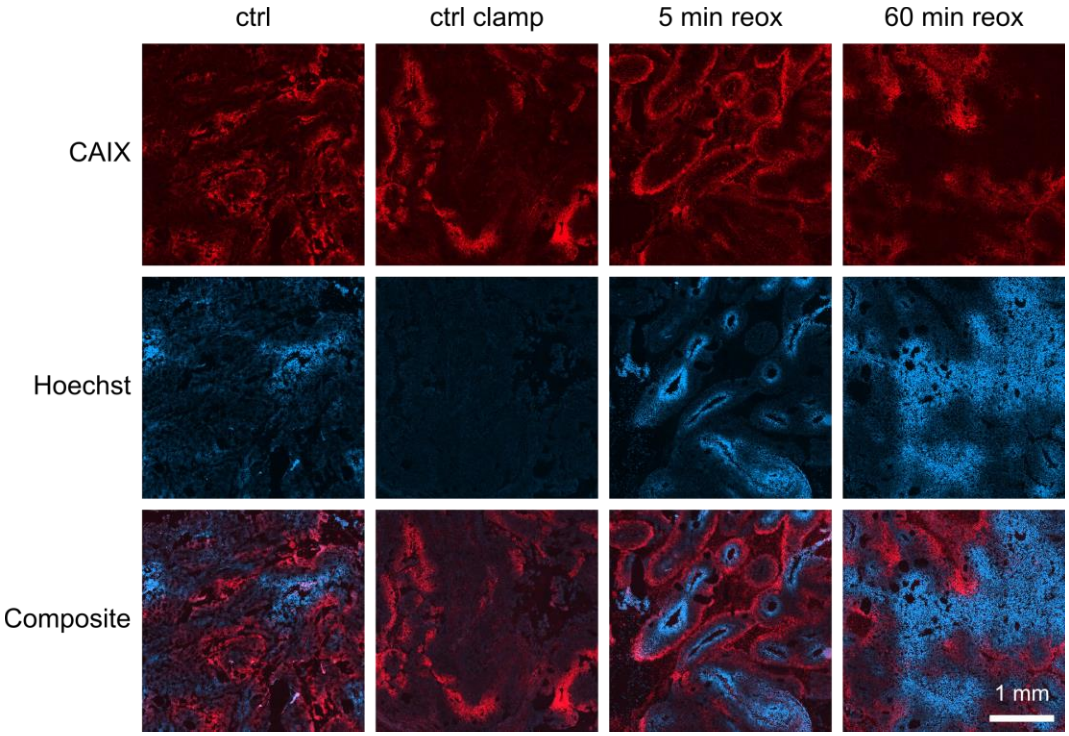

Supplementary figure 1: Immunofluorescence staining of CAIX (red) and Hoechst (blue).

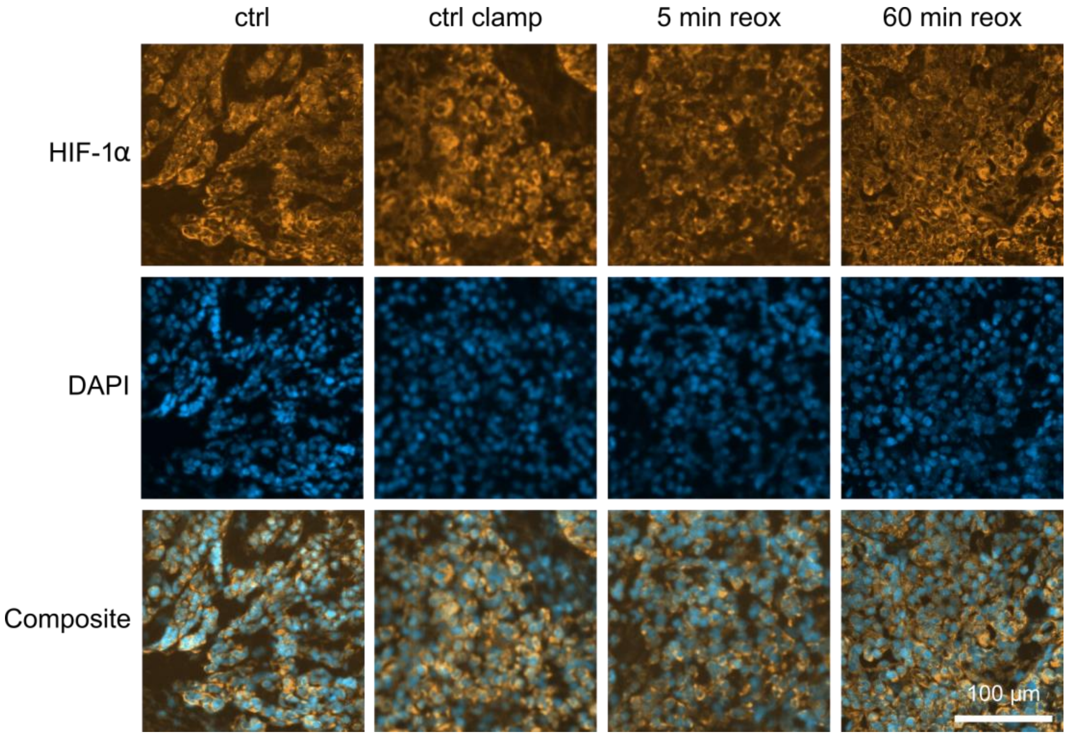

Supplementary figure 2: Immunofluorescence staining of HIF-1α (orange) and DAPI (blue).

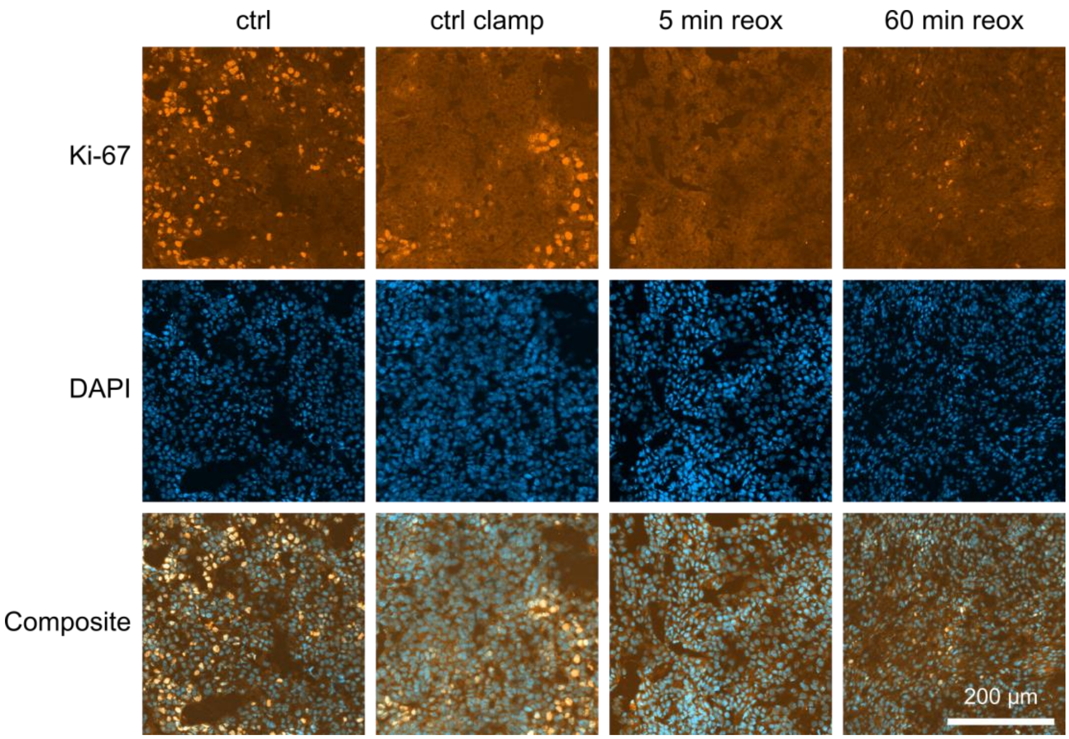

Supplementary figure 3: Immunofluorescence staining of Ki-67 (orange) and DAPI (blue).
